# Aberrant DNA hypermethylation-silenced SOX21-AS1 gene expression and its clinical importance in oral cancer

**DOI:** 10.1186/s13148-016-0291-5

**Published:** 2016-11-26

**Authors:** Cheng-Mei Yang, Tsung-Han Wang, Hung-Chih Chen, Sung-Chou Li, Ming-Chien Lee, Huei-Han Liou, Pei-Feng Liu, Yu-Kai Tseng, Yow-Ling Shiue, Luo-Ping Ger, Kuo-Wang Tsai

**Affiliations:** 1 Department of Stomatology, Kaohsiung Veterans General Hospital, Kaohsiung, Taiwan; 2 Department of Dental Technology, Shu-Zen Junior College of Medicine and Management, Kaohsiung, Taiwan; 3 Department of Medical Education and Research, Kaohsiung Veterans General Hospital, Kaohsiung, 813 Taiwan; 4 Institute of Biomedical Sciences, National Sun Yat-Sen University, Kaohsiung, Taiwan; 5 Genomics and Proteomics Core Laboratory, Department of Medical Research, Kaohsiung Chang Gung Memorial Hospital and Chang Gung University College of Medicine, Kaohsiung, Taiwan; 6 Department of Anesthesiology, Kaohsiung Veterans General Hospital, Kaohsiung, Taiwan; 7 Department of Biotechnology, Fooyin University, Kaohsiung, Taiwan; 8 Department of Orthopedics, Show Chwan Memorial Hospital, Changhua, Taiwan; 9 Department of Orthopedics, National Cheng Kung University Hospital, Tainan, Taiwan; 10 Department of Chemical Biology, National Pingtung University of Education, Pingtung, Taiwan

**Keywords:** SOX21, SOX21-AS1, Next-generation sequencing, LncRNA, Oral cancer, DNA methylation

## Abstract

**Background:**

Long noncoding RNAs (lncRNAs) are more than 200 nucleotides in length and lack transcriptional ability. The biological function of lncRNAs in oral squamous cell carcinoma (OSCC) remains unclear. The aim of this study was to identify the dysfunction of lncRNA in OSCC.

**Results:**

We analyzed the transcriptome profiles of human OSCC tissues and paired adjacent normal tissues from two patients through a next-generation sequencing approach. A total of 14 lncRNAs were upregulated (fold change ≥3) and 13 were downregulated (fold change ≤−3) in OSCC tissues compared with the adjacent normal tissues. SOX21-AS1 was subjected to further analysis, revealing that the expression levels of SOX21-AS1 significantly decreased in OSCC compared with the adjacent normal tissue. The promoter activity of SOX21-AS1 was obviously suppressed by in vitro methylation. The DNA methylation status of the SOX21-AS1 promoter was analyzed using combined bisulfite restriction analysis, revealing that the aberrant promoter hypermethylation of SOX21-AS1 was observed frequently in OSCC tissues. The effects of SOX21-AS1 on cell proliferation and invasion were examined through transient transfection. Our data showed that SOX21-AS1 could significantly suppress oral cancer cell growth and invasion. Furthermore, the low expression level of SOX21-AS1 was significantly correlated with an advanced stage (*P* = 0.047), large tumor size (*P* = 0.033), and poor disease-specific survival in OSCC patients (*P* = 0.002).

**Conclusions:**

SOX21-AS1 was identified as susceptible dysfunction correlated with promoter hypermethylation in OSCC. Low SOX21-AS1 expression may be an adverse prognostic biomarker for OSCC.

**Electronic supplementary material:**

The online version of this article (doi:10.1186/s13148-016-0291-5) contains supplementary material, which is available to authorized users.

## Background

Oral cancer is one of the most common cancers in developing countries and occurs in the anterior tongue, cheek, floor of the mouth, gingiva, and other parts of the oral cavity [1]. Oral squamous cell carcinoma (OSCC) is the most common oral cancer and 300,400 incident cases and 145,400 deaths have been reported worldwide [2]. Betel quid chewing, tobacco smoking, and alcohol drinking contribute to the increasing incidence and mortality rates of oral cancer [3].

Long noncoding RNAs (lncRNAs) are over 200 nucleotides in length and lack transcriptional ability [4]. Some lncRNAs, such as transfer RNAs (tRNAs), ribosomal RNAs (rRNAs), and spliceosomal RNAs, are crucial for maintaining normal cellular mechanisms [5]. Except for these functional lncRNAs, most lncRNAs have been frequently considered functionless sequences [6–8]. Recently, lncRNAs have been reported as crucial for regulating cell development, growth, cell cycles, and cancer metastasis in human cancer [9]. Several aberrant lncRNAs were identified from high-throughput profiles and were associated with OSCC progression [10]. Fang et al. reported that urothelial cancer associated 1 (*UCA1*) is an overexpression in tongue squamous cell carcinomas (TSCCs) and may play an oncogenic role in tumorigenesis [11]. Jia et al. [12] reported that the loss of miR-26a expression resulted in MEG3 downregulation in TSCC tissues compared with adjacent normal tissues. Low MEG3 expression was highly correlated with poor prognosis of patients. Furthermore, MEG3 overexpression in SCC-15 and CAL27 cells inhibited cell proliferation and cell cycle progression and promoted apoptosis [12]. Oncogenic lncRNA, HOX transcript antisense RNA (HOTAIR), was significantly upregulated in OSCC, and the upregulated expression was associated with poor survival and metastasis. HOTAIR knockdown can suppress oral cancer cell proliferation, colony formation, cell migration, and invasion [13]. However, the role of lncRNA remains largely unknown, and the detailed mechanisms and functions of dysfunctional lncRNAs in OSCC have not yet been fully elucidated. On the basis of the analysis of the expression profile of two pairs of OSCC tissue samples, as well as the experimental studies of two OSCC cell lines and 86 pairs of OSCC tissue samples in this study, we determined that SOX21-AS1 expression was silenced with an aberrant methylation promoter; moreover, the low expression was highly correlated with poor prognosis of patients with OSCC.

## Results

### Long noncoding RNAs identified in OSCC tissues through next-generation sequencing

**Fig. 1:**
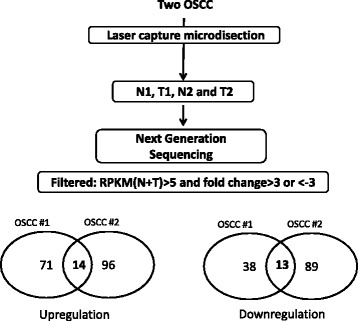
Flowchart for identifying abnormal lncRNA candidates through NGS. The OSCC specimens from two patients were stained using H&E staining. Normal and OSCC tissues were carefully separated and collected through microdissection. Total RNAs were extracted, and the transcriptome profiles were analyzed using NGS. Comparison of the lncRNA expression profiles between the tumor and normal samples in the filtering steps are as follows: (1) fold change ≥3 or <−3 and (2) sums of RPKM in normal and tumor tissues ≥5. Venn diagrams depicting the number of upregulated and downregulated lncRNA candidates in two paired samples of OSCC

Transcriptome profiles were comprehensively analyzed on laser capture microdissected tumors and corresponding adjacent normal tissues from two OSCC patients through a next-generation sequencing (NGS) approach. More than 30 million clean reads were identified, and >84% clean reads were mapped to human reference genes in four individual libraries. After the clean reads were mapped to the genome, over 560 expressed lncRNA genes were detected in four individual libraries (reads per kilobase per million [RPKM] > 1) (Additional file 1: Table S1). Dysregulated lncRNAs were selected from two OSCC patients and were highly expressed (RPKM of normal + RPKM of tumor > 5), and the fold change of RPKM was >3 or <−3 as those of tumor tissues compared with normal tissues. Our data revealed that 109 lncRNAs were dysregulated (fold change ≥3 or <−3) in tumor tissues compared with normal tissues from patient 1; among these lncRNAs, 71 and 38 were upregulated and downregulated, respectively. In addition, 185 lncRNAs were differentially expressed in patient 2; 96 and 89 of these lncRNAs were upregulated and downregulated, respectively. Combining the two profiles revealed that 14 lncRNAs were significantly upregulated (fold change ≥3), and 13 lncRNAs were downregulated (fold change ≤−3) in OSCC tissues compared with adjacent normal tissues (Fig. 1 and Table 1).

**Table 1:** LncRNA candidates were dysregulation in OSCC

LncRNA	N1 (RPKM)	T1 (RPKM)	Fold change (T1/N1)	N2 (RPKM)	T2 (RPKM)	Fold change (T2/N2)
Upregulation						
LINC00152	1.73	7.26	4.12	7.89	117.34	14.87
LOC102724332	1.66	331.85	199.91	0.1	18.3	183
LOC102724977	1.12	161.33	144.04	0.53	8.18	15.43
LOC100507420	0.66	6.77	10.26	0.35	5.35	15.29
LOC102546229	0.1	5.8	58	3.15	46.02	14.61
LOC105379353	1.45	7.61	5.25	2.34	27.73	11.85
LOC654342	2.99	12.74	4.26	1.44	16.05	11.15
LOC105370202	1.09	39.99	36.69	0.63	6.62	10.51
LOC101927797	3.44	13.56	3.94	3.58	20.47	5.72
LOC105373098	0.52	21.34	41.04	3	16.65	5.55
LOC105376387	0.57	5.37	9.42	2.07	10.84	5.24
LOC642934	1.46	15.99	10.95	10.4	40.97	3.94
LOC105376510	1.04	7.78	7.48	17.84	58.28	3.27
LOC105378451	0.29	8.43	29.07	1.77	5.74	3.24
Downregulation						
SOX21-AS1	4.47	0.78	0.17	5.43	0.11	0.02
LOC102724112	7.11	2.24	0.32	5.26	0.95	0.18
LOC105379155	9.68	0.84	0.09	7.08	1.1	0.16
LOC105371435	36.74	5.75	0.16	8	0.75	0.09
LOC101928844	14.09	0.92	0.07	11.1	0.84	0.08
LOC105374232	3.69	0.55	0.15	6.24	0.45	0.07
LOC105378948	12.78	3.85	0.30	18.49	1.07	0.06
LOC105379031	4.52	0.35	0.08	5.3	0.1	0.02
LOC105371446	24.63	0.87	0.04	13.02	0.1	0.01
LOC105378132	58.25	2.26	0.04	16.6	0.1	0.01
LOC441178	146.13	5.31	0.04	17.3	0.1	0.01
LOC105372164	15.27	3.76	0.25	19.72	0.1	0.01
LOC101928815	11.7	2.55	0.22	17.15	0.02	0.001

### DNA methylation-silenced SOX21-AS1 expression in oral cancer cells

**Fig. 2:**
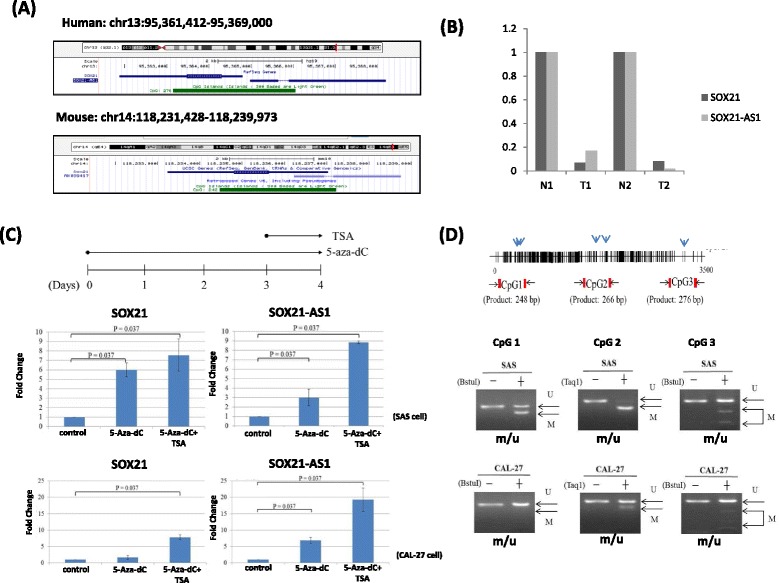
DNA methylation regulated expression of SOX21 and SOX21-AS1 in oral cancer cells. **a** Schema of the locations of the SOX21 and SOX21-AS1 genes from using the UCSC Genome Browser of the human genome GRCh37/hg19 (*upper panel*) and mouse genome GRCm38/mm10 (*lower panel*). *Green blocks* indicate the upstream CpG islands. **b** SOX21 and SOX21-AS1 expression was obtained from the NGS data. **c** SAS and CAL27 cells were treated with 10 μM 5-Aza-dC for 4 days, and 0.25 μM TSA was added on the third day (*upper panel*). After the cells were treated with 5-Aza-dC and TSA, SOX21 and SOX21-AS1 expression was examined using real-time PCR (*lower panel*). **d** Schema of the map of the CpG-rich region of SOX21-AS1. CpG sites are indicated with vertical ticks, and BstU1 or TaqI restriction sites are indicated with *vertical arrows*, in which the *arrowheads* indicate the primers used for analyzing the methylation status of CpG islands (CpG1 248 bp, CpG2 266 bp, and CpG3 276 bp), obtained through COBRA in this study (*upper panel*). DNA methylation status of CpG islands in SAS and CAL27 cells were examined using the COBRA assay. *Arrows* indicate the unmethylated (*u*)/methylated (*m*) alleles (*lower panel*)

Our previous studies have mainly focused on DNA methylation regulating noncoding RNA expression in human cancer [14–17]. Therefore, we sought to determine whether DNA hypermethylation silences lncRNA expression in oral cancer. Because a CpG-rich region is located upstream of SOX21-AS1, we investigated SOX21-AS1 further. SOX21-AS1 is a 2986-bp long noncoding RNA, which shares a bidirectional promoter with SOX21 at human chromosome 13q32.1. SOX21 and SOX21-AS1 share a head-to-head promoter, which is also conserved in the mouse genome. Furthermore, a CpG-rich region is located upstream of *SOX21-AS1* and *SOX21* (Fig. 2a), implying that their transcriptional activity may be controlled through DNA methylation. Our profiles revealed that *SOX21* and *SOX21-AS1* are simultaneously downregulated in OSCC tissues compared with paired adjacent normal tissues (Fig. 2b). Mitchell et al. reported that methylation of the sense *SOX21* was markedly lower in nonneoplastic colorectal tissues than in colorectal tumors and adenomas [18]. Therefore, we investigated whether *SOX21* and *SOX21-AS1* were silenced by aberrant DNA methylation in OSCC cell lines. After 5-Aza-dC treatment, SOX21-AS1 and SOX21 expression was increased in the SAS and CAL27 cells (Fig. 2c). The coordinated effects of histone acetylation and DNA methylation were further examined. Our data showed synergistic effects on the increases of SOX21-AS1 and SOX21 expression in the SAS and CAL27 cells with TSA and 5-Aza-dC cotreatment (Fig. 2c). In addition, a DNA methylation assay revealed DNA methylation of the CpG-rich region upstream of SOX21-AS1 in two oral cancer cell lines (Fig. 2d). Consequently, TSA and a DNA demethylating reagent caused marked synergistic activation of SOX21 and SOX21-AS1 genes in oral cancer cells.

**Fig. 3:**
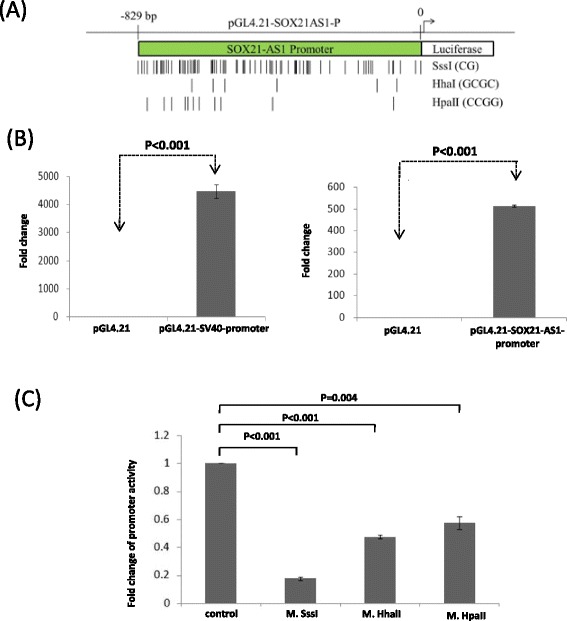
Promoter activity of SOX21-AS1 was silenced using in vitro methylation. **a** Schema of the luciferase constructs containing the putative promoter of SOX21-AS1 (pGL4.21-SOX21-AS1-P 0–829 bp). **b** Promoter constructs were transfected into SAS cells for 24 h, and luciferase activity was examined using the Dual-Glo luciferase reporter assay system kit. The SV40 promoter was the positive control. Relative luciferase activity was compared using the ratio of *Renilla reniformis* and firefly luciferase activity. **c** pGL4.21-SOX21-AS1-P was methylated in vitro by using M. SssI, M. HpaII, and M. HhaI methylase enzymes. The promoter activity was examined using the luciferase reporter assay

To further confirm whether SOX21-AS1 expression was silenced by DNA methylation, we assessed the promoter activity of SOX21-AS1 through an in vitro methylation assay. As shown in Fig. 3a, b, the activity of the PGL-SOX21-AS1 promoter significantly increased (>500-fold) compared with that of the negative control, implying that this DNA fragment exhibited promoter activity. The pGL-SOX21-AS1 promoter luciferase activity was almost entirely repressed after complete methylation of all CpG dinucleotides by M. SssI. However, the promoter constructs of SOX21-AS1 were partially methylated by M. HhaI or M. HpaII, revealing that the promoter activity of SOX21-AS1 was only partially suppressed compared with that of the control groups (Fig. 3c). The luciferase activity of two CpG dinucleotide-less promoter constructs, pGL-SRE and pGL-CRE, were not repressed after complete methylation of all CpG dinucleotides by M. SssI (Additional file 2: Figure S1). Thus, these data revealed that the transcriptional activity of the putative promoter of SOX21-AS1 can be repressed using DNA hypermethylation in oral cancer cells.

### DNA hypermethylation results in SOX21-AS1 silencing in OSCC

**Fig. 4:**
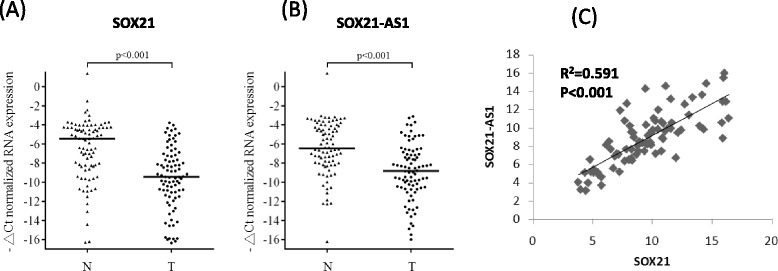
SOX21 and SOX21-AS1 expression was silenced using DNA methylation. **a**, **b** SOX21 and SOX21-AS1 expression in the 86 pairs of adjacent normal and tumor tissues examined through real-time PCR. **c** Correlation coefficients of SOX21-AS1 and SOX21 expression in OSCC

**Fig. 5:**
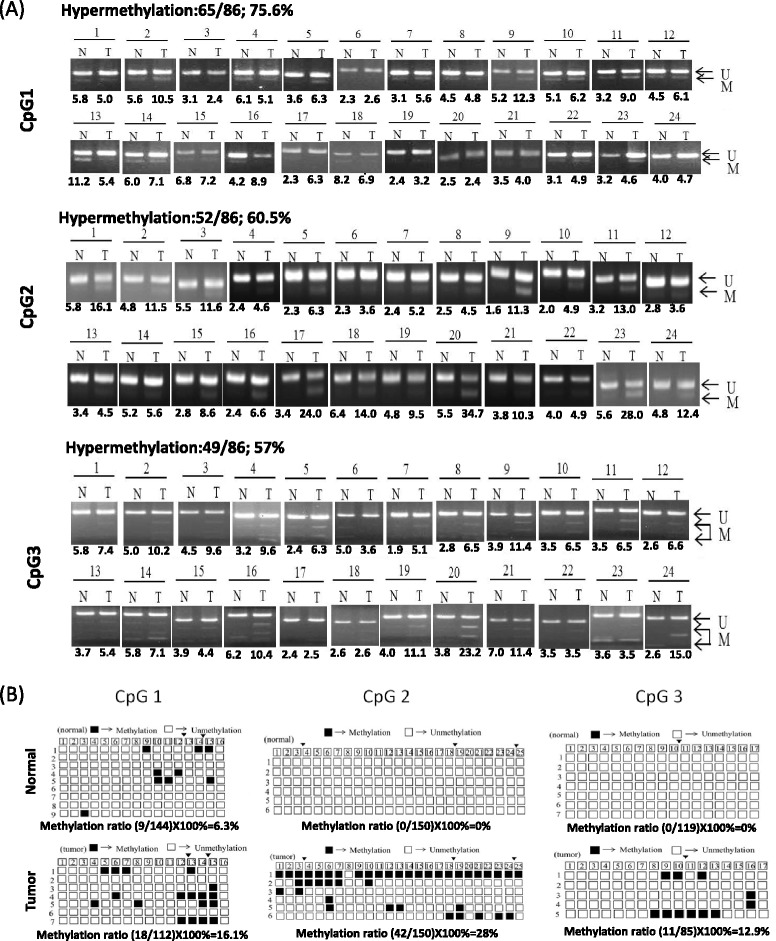
Methylation status of promoter regions of SOX21 and SOX21-AS1 in 86 patients with OSCC. **a** Methylation status of three CpG-rich regions of the SOX21-AS1 promoter was analyzed in the OSCC genome by using COBRA. *Arrows* indicate the unmethylated (*u*)/methylated (*m*) alleles. We quantified the DNA fragments by using ImageJ, and the DNA methylation status was calculated according to the digested fragment/(undigested fragment + digested fragment) (*lower panels*). **b** Methylation status of one patient (patient 9) examined using bisulfite sequencing. Each *row* represents a single clone for each PCR product. *Open* and *filled squares* represent unmethylated and methylated CpG sites, respectively

Our data revealed that SOX21 and SOX21-AS1 expression was significantly reduced in OSCC tissues compared with adjacent normal tissues (SOX21: 62 of 86, *P* < 0.001; SOX21-AS1: 61 of 86, *P* < 0.001). Simultaneous downregulation of SOX21 and SOX21-AS1 was quite frequent in the 86 OSCC samples (60.5%; 52 of 86; Fig. 4a, b). Thus, SOX21 expression was significantly correlated with SOX21-AS1 expression in 86 OSCC tissues (*R* ^2^ = 0.591, *P* < 0.001, Fig. 4c). Previous studies have reported that combined bisulfite restriction analysis (COBRA) is well suited for analyzing numerous clinical specimens [19]. Therefore, we further examined the methylation status of the SOX21-AS1 promoter region by using a COBRA assay. Our data revealed frequent DNA methylation in OSCC tissues (CpG1 75.6%, CpG2 60.5%, and CpG3 57%; Fig. 5a). Tumor-specific hypermethylation of CpG region 2 was frequently observed in oral cancer (36 of 86, 41.9%) and resulted in decreased SOX21-AS1 expression in OSCC tissues compared with adjacent normal tissues (25 of 36, 69.4%). In addition, the high methylation status of CpG region 2 also correlated with low SOX21-AS1 expression, but CpG regions 1 and 3 were not thus correlated (Additional file 3: Figure S2). Subsequent bisulfite sequences of three CpG-rich regions in the selected patient (patient 9) were consistent with those of the COBRA data (Fig. 5b). Thus, our data revealed that SOX21 and SOX21-AS1 expression can be silenced using a DNA-methylated promoter in oral cancer.

### SOX21-AS1 suppresses oral cancer cell growth and invasion

**Fig. 6:**
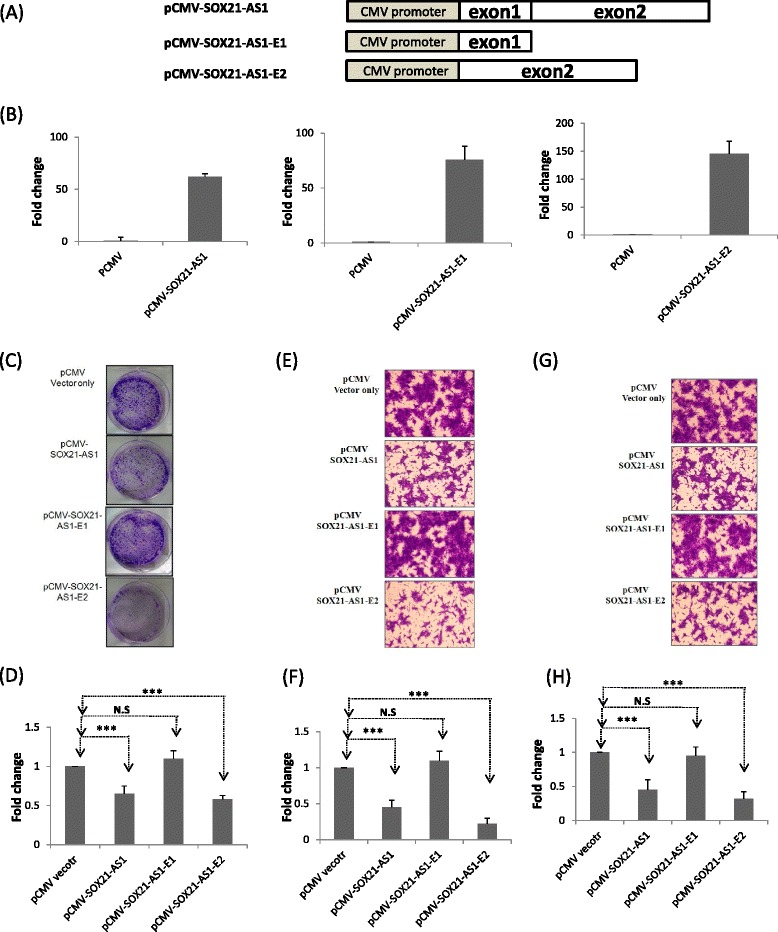
Ectopic SOX21-AS1 expression affected cell growth in oral cancer cells. **a** Schema of the expression constructs (pCMV-SOX21-AS1, pCMV-SOX21-AS1-exon1, and pCMV-SOX21-AS1-exon2, respectively). **b** Relative levels of SOX21-AS1, exons 1 and 2, in the SAS cells were analyzed using real-time PCR after transiently transfecting pCMV, pCMV-SOX21-AS1, pCMV-SOX21-AS1-exon1, and pCMV-SOX21-AS1-exon2, sequentially. **c**, **d** Colony formation assays were used to examine the SAS cell colonies after transient transfection with the expression constructs for 2 weeks. Cell photographs of a representative experiment are shown, and the graph was quantified using Ascent. Data are reported as colonies compared with control (means ± SD). **e**–**h** The migration and invasion ability were assessed using the transwell assay in SAS cells. After transfection for 24 h, cells were subjected to the transwell assay for 48 h. Cells were then stained with crystal violet solution, and the numbers of migrating cells were quantified by counting three fields under a phase-contrast microscope

We analyzed the subcellular localization of endogenous SOX21-AS1, revealing SOX21-AS1 expression accumulating in the nuclei of SAS cells (Additional file 4: Figure S3A). The predominantly nuclear localization of SOX21-AS1 might escape RNA interference-silencing machinery, which is the main action in the cytoplasms of the cells. Therefore, gain-of-function might be a more effective approach to determining the biological function of SOX21-AS1. We constructed a SOX21-AS1 expression vector consisting of two exons with a total length of approximately 2.9 kb. The putative biological function of SOX21-AS1 was examined through transient transfection of pCMV-SOX21-AS1 into SAS cell lines. After transfection, the SOX21-AS1 expression levels were significantly overexpressed (>60-fold increase) compared with the vector control group (Fig. 6a, b). Overexpression of SOX21-AS1 in SAS cells could significantly inhibit cancer cell growth, migration, and invasion (Fig. 6c–h). However, ectopic SOX21-AS1 expression did not influence endogenous SOX21 expression, implying that SOX21-AS executes its biological function through an SOX21-independent pathway (Additional file 4: Figure S3B).

We made two additional deletion constructs, pCMV-SOX21-AS1-E1 and pCMV-SOX21-AS1-E2, for determining which region is responsible for cell growth and motility inhibition. As shown in Fig. 6a, b, both SOX21-AS1-E1 and SOX21-AS1-E2 were significantly overexpressed (>80-fold increase) compared with the vector control group. The effects of SOX21-AS1-E1 and SOX21-AS1-E2 on the proliferation, migration, and invasion of SAS cells were then respectively examined after transient transfection. Our data revealed that ectopic overexpression of SOX21-AS1-E1 revealed no effect on proliferation or motility ability in SAS cells. Ectopic SOX21-AS1-E2 expression in SAS cells significantly suppressed the growth and motility ability of SAS cells (Fig. 6c–h). To understand the putative mechanism of SOX21-AS1 suppressed cell growth and invasion ability, we further examined the epithelial-mesenchymal transition (EMT) genes and growth-related genes. As shown in Additional file 4: Figure S3C, the expression level of E-cadherin was increased, and fibronectin and cyclin D1 were decreased in SAS cells with SOX21-AS1 overexpression. However, the details of the mechanisms of SOX21-AS1 suppressed cell growth and invasive ability remain unclear and require further study.

### Correlation between SOX21-AS1 expression and clinicopathological characteristics

The effects of SOX21 and SOX21-AS1 expression on the clinicopathologic outcomes of patients were evaluated, revealing that the low SOX21-AS1 expression was significantly correlated with the pathological stage (*P* = 0.047) and T classification (*P* = 0.033, Table 2). However, no correlation was observed between SOX21 expression and clinicopathologic features. We further evaluated the association of SOX21-AS1 expression with survival in OSCC patients. Kaplan–Meier curves and the Cox regression analysis revealed that low SOX21-AS1 expression was significantly associated with a short disease-specific survival (log-rank *P* = 0.013, Additional file 5: Figure S4 and Table 3). The multivariate Cox regression model revealed that low SOX21-AS1 expression was significantly correlated with shorter disease-specific survival and disease-free survival for patients with OSCC (DSS: adjusted hazard ratio 5.66, 95% CI 1.85–17.30, *P* = 0.002; DFS: adjusted hazard ratio 2.96, 95% CI 1.14–7.64, *P* = 0.025, Table 3), after adjustment for cell differentiation, pathological stages, and the expression level of SOX21.

**Table 2:** The relationship of expression levels of SOX21 and SOX21-AS1 with clinicopathologic data of OSCC patients

Variables	SOX21 (*n* = 86)			SOX21-AS1 (*n* = 86)		
	Low expression	High expression	*P* value	Low expression	High expression	*P* value
	Number (%)	Number (%)		Number (%)	Number (%)	
Sex						
Female	5 (55.6)	4 (44.4)	1.000^a^	2 (22.2)	7 (77.8)	0.712^a^
Male	38 (49.4)	39 (50.6)		27 (35.1)	50 (64.9)	
Age						
≤50	12 (54.5)	10 (45.5)	0.621^b^	8 (36.4)	14 (63.6)	0.761^b^
>50	31 (48.4)	33 (51.6)		21 (32.8)	43 (67.2)	
Cancer location						
Buccal and other oral mucosal sites	36 (54.5)	30 (45.5)	0.126^b^	25 (37.9)	41 (62.1)	0.138^b^
Tongue	7 (35.0)	13 (65.0)		4 (20.0)	16 (80.0)	
Cell differentiation						
Well	5 (41.7)	7 (58.3)	0.534^b^	4 (33.3)	8 (66.7)	1.000^a^
Moderate, poor	38 (51.4)	36 (48.6)		25 (33.8)	49 (66.2)	
AJCC pathological stage						
I	6 (37.5)	10 (62.5)	0.268^b^	2 (12.5)	14 (87.5)	*0.047* ^b^
II, III, IV	37 (52.9)	33 (47.1)		27 (38.6)	43 (61.4)	
T classification						
T1	6 (35.3)	11 (64.7)	0.176^b^	2 (11.8)	15 (88.2)	*0.033* ^b^
T2, T3, T4	37 (53.6)	32 (46.4)		27 (39.1)	42 (60.9)	
N classification						
N0	32 (50.0)	32 (50.0)	1.000^b^	20 (31.2)	44 (68.8)	0.683^b^
N1, N2	11 (50.0)	11 (50.0)		9 (40.9)	13 (59.1)	

The italic indicated *p* value <0.05

^a^ *P* value was estimated by Fisher’s exact test

^b^ *P* value was estimated by chi-square test

**Table 3:** Univariate and multivariate Cox’s regression analysis of SOX21-AS1 expression for disease-specific survival and disease-free survival of OSCC patients

Variable	Number (%)	Number of events	Univariate analysis			Multivariate analysis		
			Hazard ratio	95% CI	*P* value	Hazard ratio	95% CI	*P* value^a^
Disease-specific survival								
SOX21-AS1								
High	57 (66.3)	8	1.00			1.00		
Low	29 (33.7)	10	3.07	1.21–7.78	*0.018*	5.66	1.85–17.30	*0.002*
Disease-free survival								
SOX21-AS1								
High	57 (66.3)	16	1.00			1.00		
Low	29 (33.7)	10	1.60	0.72–3.54	0.249	2.96	1.14–7.64	*0.025*

The italic indicated *p* value <0.05

^a^ *P* values were estimated by multiple Cox’s regression, in which adjusted for cell differentiation (moderate and poor vs well), AJCC pathological stage (stages III and IV vs stages I and II), and SOX21 (high vs low)

## Discussion

After the entire human genome was completely sequenced in 2003, it was revealed that only approximately 2% of the human genome is used for protein coding [20]. High-throughput sequencing data revealed that 80–90% of the human genomic DNA is actively produced by RNA transcripts [20–22]. Most of these transcripts are nonprotein coding genes, including small RNAs and lncRNAs. In the present study, we comprehensively analyzed OSCC profiles by using an NGS approach, which revealed that the expression of 27 lncRNAs significantly differed between OSCC and the corresponding adjacent normal tissues (Table 1). Among lncRNAs, lnc00152 has been reported to play an oncogenic role in promoting gastric cancer cell growth, migration, and invasion and in suppressing cell apoptosis by sponging miR-18a-5p, miR-195-3p, miR-139-5p, and miR-31-5p expression [23, 24]. In addition, lnc00152 was significantly overexpressed in pancreatic cancer [25]. However, in-depth biological function and the effect of dysfunctional lnc00152 on oral cancer remain unclear and require further investigation.

In this study, our data revealed that SOX21 and SOX21-AS1 share a head-to-head promoter, and DNA methylation of the promoter leads to simultaneous suppression of their expression in OSCC. Our data showed that SOX21-AS1 expression was increased in oral cancer cells treated with 5-Aza-dC. An in vitro methylation assay demonstrated that SOX21-AS1 promoter activity could be suppressed with complete DNA methylation. However, only the methylation status of CpG2 was well correlated with low SOX21-AS1 expression in OSCC, but CpG regions 1 and 3 were not thus correlated (Additional file 3: Figure S2). These conflicting results may be due to two putative reasons: (1) A COBRA assay does not completely represent DNA methylation status; (2) DNA methylation is not the only factor for transcriptional silencing.

A previous study revealed that SOX21 expression was controlled through DNA methylation and was a suitable biomarker for colorectal cancer diagnosis [18]. In human glioma cells, SOX21 overexpression inhibits SOX2 expression and induces apoptosis because of the interaction between SOX21 and SOX2 [26, 27]. Ferletta et al. reported that the knockdown of SOX2 expression can suppress SOX21 expression at the protein and mRNA levels in glioma cells, implying that SOX2 can positively regulate the transcriptional activity of SOX21 [27]. SOX21 in combination with SOX2 exerts a tumor-suppressive effect on carcinogenesis. Although SOX21 and SOX21-AS expression were reduced simultaneously in OSCC, SOX21 expression does not contribute to the clinical manifestations of patients with OSCC (Tables 2 and 3). Conversely, low SOX21-AS1 expression was significantly correlated with poor clinicopathologic features and a short survival time of patients with OSCC (Tables 2 and 3).

## Conclusions

Although SOX21-AS1 can suppress oral cancer cell growth and invasion, the details of the underlying mechanism remain unknown. LncRNAs can modulate cellular function through different mechanisms such as RNA degradation, epigenetic modification, transcriptional regulation, and an miRNA decoy [28]. We analyzed the subcellular fractionation localization of SOX21-AS1 in SAS cells (Additional file 4: Figure S3). Furthermore, a considerable increase in SOX21-AS1 expression was observed in the nuclei compared with the cytoplasms. According to its location and tumor-suppressive role, SOX21-AS1 is probably an enhancer that reinforces other tumor suppressor mRNAs or acts as a decoy removing certain transcription factors from binding to the DNA to prevent oncogene transcription. In conclusion, low SOX21-AS1 expression correlated with its sense gene SOX21 and can serve as an independent biomarker for poor prognosis of OSCC. SOX21-AS1 and SOX21 expression in tumors may be downregulated through DNA hypermethylation. However, the details of the biological functions of SOX21-AS1 must be further evaluated in OSCC.

## Methods

### Clinical samples

Clinical tissue samples were collected from OSCC patients who provided signed informed consent and accepted a surgical operation at the Department of Dentistry and Department of Otorhinolaryngology, Kaohsiung Veterans General Hospital. This study protocol was approved by the Institutional Review Board of Kaohsiung Veterans General Hospital (Kaohsiung, Taiwan; IRB number VGHKS14-CT6-18). The total RNA and DNA of the tissues were extracted using the TRIzol reagent (Invitrogen, Carlsbad, CA, USA) according to the instruction manual.

### Next-generation sequencing and analysis

In this study, the transcriptome profiles of paired tumors and adjacent normal tissue samples from two OSCC patients were analyzed using an NGS approach; the detailed clinical characteristics of the samples are shown in Additional file 6: Table S2. High-purity normal and tumor tissues were individually separated through laser capture microdissection and were transferred to an RNA extraction kit (Qiagen, Hilden, Germany) for RNA extraction. All procedures of the RNA transcriptome were performed according to the manufacturer protocol from Illumina. Library construction of all samples was performed using TruSeq RNA Sample Prep Kits v2 for 50 single-end base pair sequencing on the Solexa platform. Finally, raw sequences were obtained from the Illumina GA Pipeline software CASAVA version 1.8 and expected to generate 30 million reads per sample. After low-quality data were filtered, qualified reads were analyzed using TopHat/Cufflinks for estimating gene expression, which was calculated as RPKM. For differential expression analyses, CummeRbund was used for statistically analyzing the gene expression profiles. The reference genome and gene annotations were retrieved from the Ensembl database.

### Cell lines and demethylation treatment

Two human OSCC cell lines, SAS (human tongue SCC) and CAL27 (human tongue SCC) cell lines, were provided by Dr. Michael Hsiao (Genomics Research Center, Academia Sinica, Taipei, Taiwan). The oral cancer cell lines, SAS and CAL27, were cultured in Dulbecco's modified Eagle’s medium supplemented with 10% fetal bovine serum (Invitrogen, Carlsbad, CA, USA) and 1% penicillin/streptomycin (Sigma-Aldrich, St. Louis, MO, USA). To examine the role of methylation in the regulation of lncRNA expression, SAS and CAL27 cells were cultured in the presence or absence of 10 μM 5-aza-2′-deoxycytidine (5-Aza-dC) for 4 days and treated with 0.25 μM TSA on the third day.

### Reverse transcriptase polymerase chain reaction of lncRNAs

For RT-PCR, 2 μg of total RNA was reverse transcribed using random primers and SuperScript III reverse transcriptase according to manufacturer instructions (Invitrogen, Carlsbad, CA, USA). Gene expression was detected using an SYBR Green I assay (Applied Biosystems, Foster City, CA, USA), and lncRNA expression was normalized to that of GAPDH (△Ct = target lncRNA Ct-GAPDH Ct). The individual primers used in the present study were as follows: SOX21-F: GCACAACTCGGAGATCAGCA and SOX21-R: CCGGGAAGGCGAACTTGTC; SOX21-AS1-exon1-F: CCGATGGGAAACCCCCAATC and SOX21-AS1-exon1-R: AACGCTTGCTCAAGCCTCAT; SOX21-AS1-exon2-F: TCACTTACATGCGCTGCTGA and SOX21-AS1-exon2-R: GCCGCAGCATACCAAAAAGT; GAPDH-F: TGCACCACCAACTGCTTAGC and GAPDH-R: GGCATGGACTGTGGTCATGAG.

### SOX21-AS1 promoter and expression construction

The SOX21-AS1 promoter was amplified using the promoter-specific primer pairs (SOX21AS1-promoter-F: ATGAAGCTTCCATGAAGGCGTTCATGGGCCG and SOX21AS1-promoter-R: ATGAAGCTTAGAGGAAGACTCGAGAGGCAGGT). PCR products were digested with the restriction enzyme HindIII and cloned into the pGL4.21 luciferase expression vector (Promega, Madison, WI, USA). Full-length exons 1 and 2 of the SOX21-AS1 PCR products were amplified using individual primer pairs (exon1-F: ATGGAATTCTCTTCTTGGCTCCGGGCAGGGTG and exon1-R: ATGAAGCTTCTGAGCCGGTGCAGAGGGCG; exon2-F: ATGGAATTCGTTTAGGCGAGTGGAGAGTCCG, and exon2-R: ATGCTCGAGAATCTTTAGGACAAAACTGAGC). The PCR products were digested with the restriction enzyme EcoRI and cloned into the pCMV-Tag 2A vector (Stratagene, La Jolla, CA, USA).

### In vitro methylation

We sequentially methylated pGL4-SV40, pGL4-SRE promoter, pGL4-CRE promoter, and pGL4-SOX21-AS1-P in vitro by using M. SssI, M. HpaII, and M. HhaI methyltransferase enzymes (Invitrogen, Grand Island, NY, USA). A luciferase assay was performed in SAS cells by using a Dual-Glo luciferase reporter assay system kit (Promega, Madison, WI, USA) 24 h after transfection.

### Combined bisulfite restriction and sequencing analyses

DNA was subjected to bisulfite conversion by using the EZ DNA Methylation-Gold Kit (Zymo Research Corporation, Orange, CA, USA). The bisulfite-converted genomic DNA was used for methylation analysis of the promoter with the specific methylation primers. The methylation status of the genomic DNA of individual samples was examined using TaqI or BstUI digestion (New England Biolabs, MA, USA). Furthermore, the PCR products were cloned into the pJET1.2 vector (Promega, Madison, WI, USA), and several clones were used for sequencing. The individual primers used in the present study were as follows: CpG1-F: ATGAAATTTTTAATAAAATTGGAAAGGT and CpG1-R: CCAAATAAAA ACAAA AAAACCAAAC; CpG2-F: GGTTGTTTTTGGGATATTTTAATTTT and CpG2-R: CTAAAAACCCCCTTTAACACTTAAC; CpG3-F: GGAGGAGGTGGAGTTTAGGATT and CpG3-R: AAAACCACAACCAAAACAACTACA.

### Clonogenic assays

SAS cells were transfected with pCMV-SOX21-AS1, pCMV-SOX21-AS1-exon1, pCMV-SOX21-AS1-exon2, and empty vectors by using Lipofectamine® 2000 Reagent (Invitrogen, Grand Island, NY, USA). For the clonogenic assay, 2000 transfecting cells were plated onto 6-well plates for 2 weeks until substantial-sized colonies were formed. Subsequently, the colonies were analyzed using crystal violet staining.

### Cell migration and invasion assays

The migration and invasion abilities of cells were assessed in vitro by using a transwell assay. In brief, cells were resuspended at a density of 4.5 × 10^5^ in 2% fetal bovine serum and then added to the upper chamber of the transwells (Falcon, Corning Incorporated, USA) without Matrigel (BD Biosciences, MA, USA) for the migration assay or with a Matrigel coating for the invasion assay. Chambers were incubated in a CO_2_ incubator at 37 °C for 36–48 h; the remaining cells in the upper chamber were removed using cotton swabs, and the cells on the undersurface of the transwells were fixed in 10% formaldehyde solution. Cells were stained with crystal violet solution, and the numbers of cancer cells in three fields were counted under a phase-contrast microscope.

### Statistical analysis

The chi-square test, Fisher exact test, Student *t* test, ANOVA, Mann–Whitney *U* test, and Kruskal–Wallis one-way ANOVA were used to evaluate the correlation of each lncRNA expression with different oral tissues or clinicopathological parameters. For clinicopathologic outcome and survival analysis, the RNA expression levels (-delta Ct) of SOX21-AS1 were dichotomized as low expression and high expression with the cutoff value (−9.8647) based on a receiver operating characteristic (ROC) curve analysis. The clinicopathologic outcome is usually defined as the time of initial diagnosis or surgery. Disease-specific survival was measured from the time of the initial resection of the primary tumor to the date of cancer-specific death or the final follow-up. Disease-free survival was calculated from the date of the initial resection of the primary tumor to the date of recurrence or the final follow-up. In this study, the median time of follow-up was 25.68 (range, 1.00–68.27) months. The cumulative survival curves were estimated using the Kaplan–Meier method, and the survival curves were compared using the log-rank test. A Cox proportional hazards model was used for determining independent predictors of survival by using factors significant in univariate analysis as covariates; *P* < 0.05 was considered statistically significant.

## Additional files

Additional file 1: Table S1. The categories of sequence reads in the four libraries. (DOC 28 kb)
Additional file 2: Figure S1. Promoter activity of CpG dinucleotideless was examined using in vitro methylation assay. Luciferase activity of CpG dinucleotide-less promoters was analyzed through in vitro methylation. (A, B) Schema of the luciferase constructs containing two CpG dinucleotide-less promoters of pGL-serum-response element and pGL-cAMP-response element (upper panels). Promoter constructs were methylated in vitro by using M. SssI methylase enzymes, and luciferase activity was examined using the Dual-Glo luciferase reporter assay system kit. (PPT 174 kb)
Additional file 3: Figure S2. The correlation between od SOX21-AS1 expression and DNA methylation status. Correlation between SOX21-AS1 expression and DNA methylation status was examined in OSCC from 86 patients. (PPT 134 kb)
Additional file 4: Figure S3. The cellular localization of SOX21-AS1 and putative mechanism were examined in SAS cell. (A) Cell-fractioned RNA was isolated from the nuclei and cytoplasms of SAS cells. Expression levels of SOX21-AS1 were detected using RT-qPCR. (B) Expression levels of SOX21 were examined using real-time PCR after SOX21-AS1 was transfected into SAS cells. (C) Expression levels of EMT- and cell cycle-relative genes were examined using a Western blot approach in SAS cells with SOX21-AS1 overexpression. (PPT 2411 kb)
Additional file 5: Figure S4. Analysis of the prognostic significance of SOX21-AS1 expression in oral cancer. (A, B) DSS and DSF were compared according to SOX21-AS1 expression levels in oral cancer tissues. (PPT 239 kb)
Additional file 6: Table S2. The detailed clinical characteristics of two OSCC patients. (DOC 29 kb)
