# Supplementary material for: Aberrant DNA hypermethylation-silenced SOX21-AS1 gene expression and its clinical importance in oral cancer
Source: Clin Epigenetics. 2016 Nov 26;8:129. doi: 10.1186/s13148-016-0291-5 (PMC5124299; doi:10.1186/s13148-016-0291-5)
Supplement: Additional file 1: Table S1. — The categories of sequence reads in the four libraries. (DOC 28 kb) [file 13148_2016_291_MOESM1_ESM.doc]

| **Supplementary Table 1. The categories of sequence reads in the four libraries** | | | | |
| --- | --- | --- | --- | --- |
| Sample name | Total Illumina Reads | Clean read | % Percentage | Detected lncRNAs |
| N1 | 35768421 | 32295307 | 90.29 | 561 |
| T1 | 36054872 | 31544408 | 87.49 | 627 |
| N2 | 35654381 | 30163606 | 84.60 | 574 |
| T2 | 34468326 | 29711697 | 86.20 | 686 |
