# Supplementary figures and images for: Aberrant DNA hypermethylation-silenced SOX21-AS1 gene expression and its clinical importance in oral cancer
Source: Clin Epigenetics. 2016 Nov 26;8:129. doi: 10.1186/s13148-016-0291-5 (PMC5124299; doi:10.1186/s13148-016-0291-5)

## Slide 1
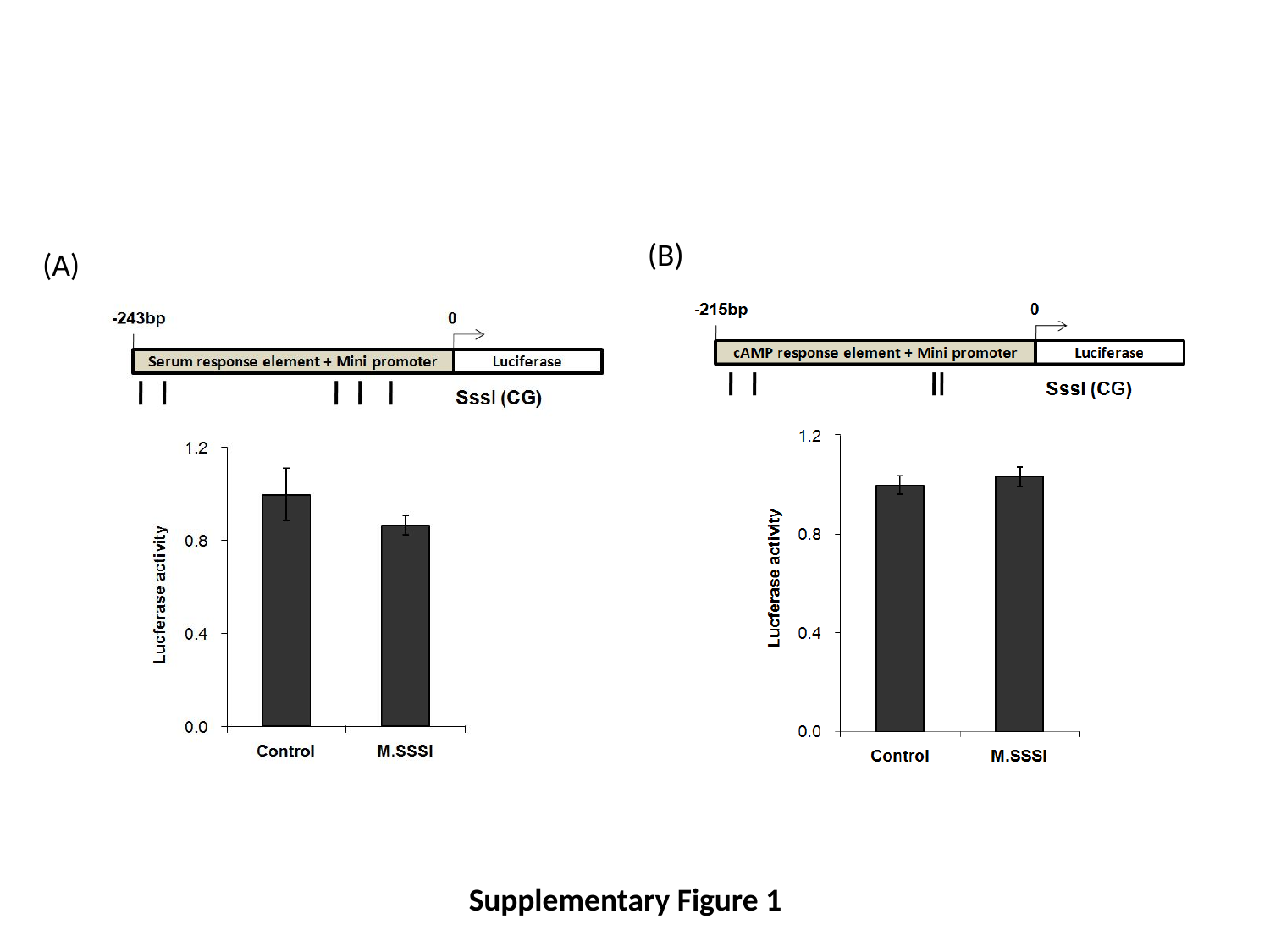

(B)
(A)
Supplementary Figure 1

Supplement: Additional file 2: Figure S1. — Promoter activity of CpG dinucleotideless was examined using in vitro methylation assay. Luciferase activity of CpG dinucleotide-less promoters was analyzed through in vitro methylation. (A, B) Schema of the luciferase constructs containing two CpG dinucleotide-less promoters of pGL-serum-response element and pGL-cAMP-response element (upper panels). Promoter constructs were methylated in vitro by using M. SssI methylase enzymes, and luciferase activity was examined using the Dual-Glo luciferase reporter assay system kit. (PPT 174 kb) [file 13148_2016_291_MOESM2_ESM.ppt]

## Slide 1
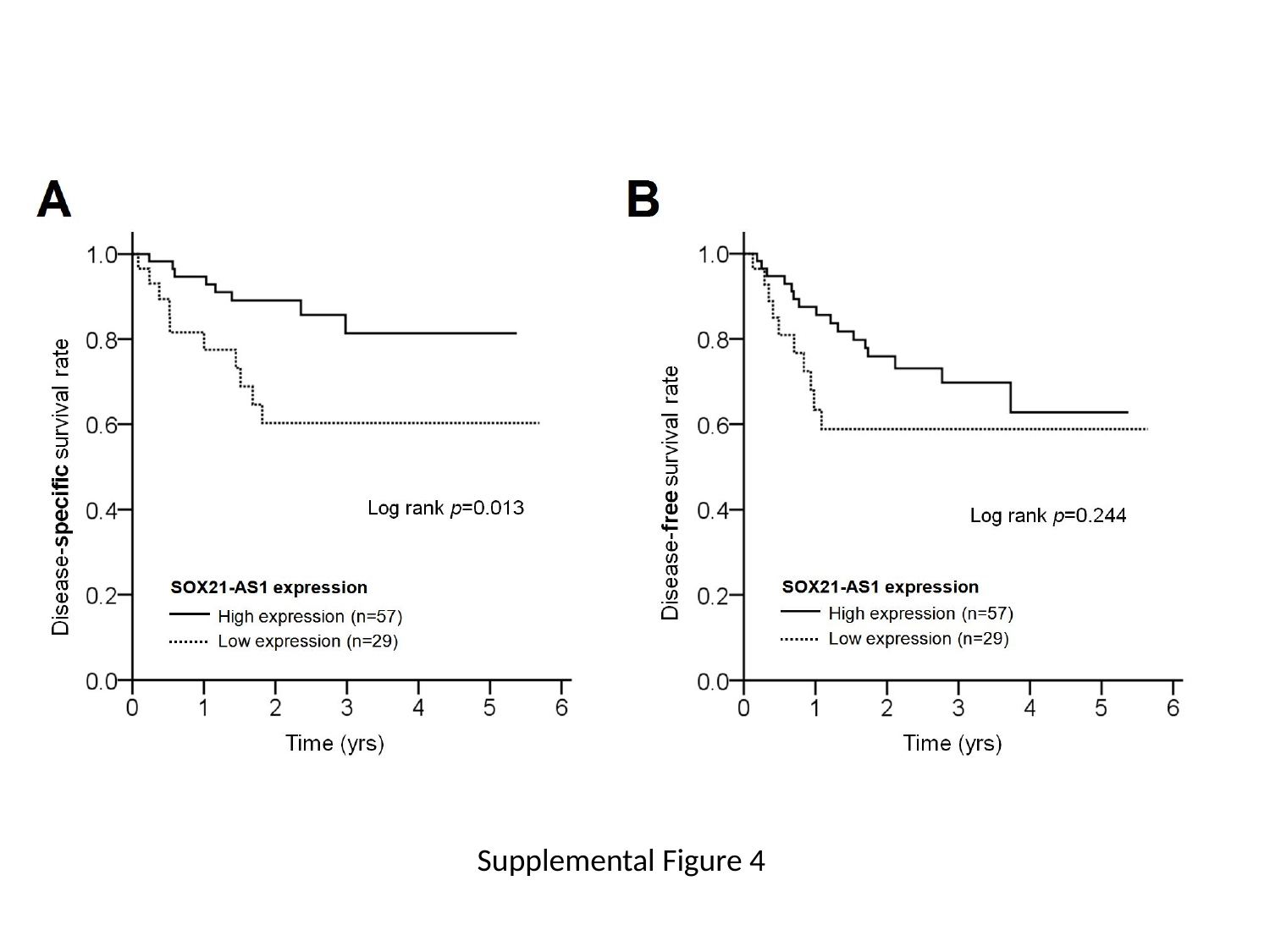

Supplemental Figure 4

Supplement: Additional file 5: Figure S4. — Analysis of the prognostic significance of SOX21-AS1 expression in oral cancer. (A, B) DSS and DSF were compared according to SOX21-AS1 expression levels in oral cancer tissues. (PPT 239 kb) [file 13148_2016_291_MOESM5_ESM.ppt]
