# Supplementary material for: Aberrant DNA hypermethylation-silenced SOX21-AS1 gene expression and its clinical importance in oral cancer
Source: Clin Epigenetics. 2016 Nov 26;8:129. doi: 10.1186/s13148-016-0291-5 (PMC5124299; doi:10.1186/s13148-016-0291-5)
Supplement: Additional file 3: Figure S2. — The correlation between od SOX21-AS1 expression and DNA methylation status. Correlation between SOX21-AS1 expression and DNA methylation status was examined in OSCC from 86 patients. (PPT 134 kb) [file 13148_2016_291_MOESM3_ESM.ppt]

## Slide 1
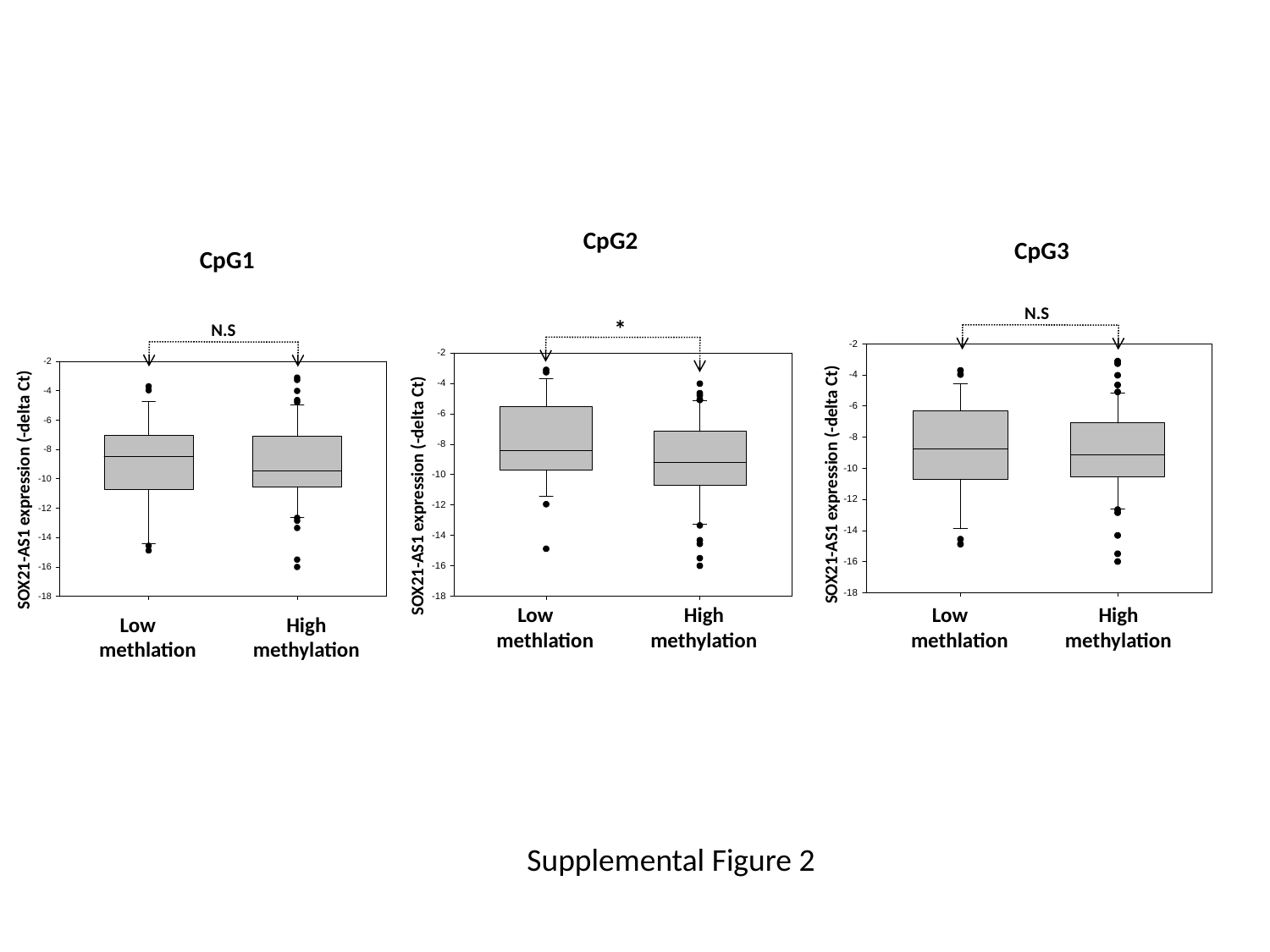

CpG2
CpG3
CpG1
N.S
*
N.S
SOX21-AS1 expression (-delta Ct)
SOX21-AS1 expression (-delta Ct)
SOX21-AS1 expression (-delta Ct)
Low methlation
High methylation
Low methlation
High methylation
Low methlation
High methylation
Supplemental Figure 2
