# Supplementary material for: Aberrant DNA hypermethylation-silenced SOX21-AS1 gene expression and its clinical importance in oral cancer
Source: Clin Epigenetics. 2016 Nov 26;8:129. doi: 10.1186/s13148-016-0291-5 (PMC5124299; doi:10.1186/s13148-016-0291-5)
Supplement: Additional file 4: Figure S3. — The cellular localization of SOX21-AS1 and putative mechanism were examined in SAS cell. (A) Cell-fractioned RNA was isolated from the nuclei and cytoplasms of SAS cells. Expression levels of SOX21-AS1 were detected using RT-qPCR. (B) Expression levels of SOX21 were examined using real-time PCR after SOX21-AS1 was transfected into SAS cells. (C) Expression levels of EMT- and cell cycle-relative genes were examined using a Western blot approach in SAS cells with SOX21-AS1 overexpression. (PPT 2411 kb) [file 13148_2016_291_MOESM4_ESM.ppt]

## Slide 1
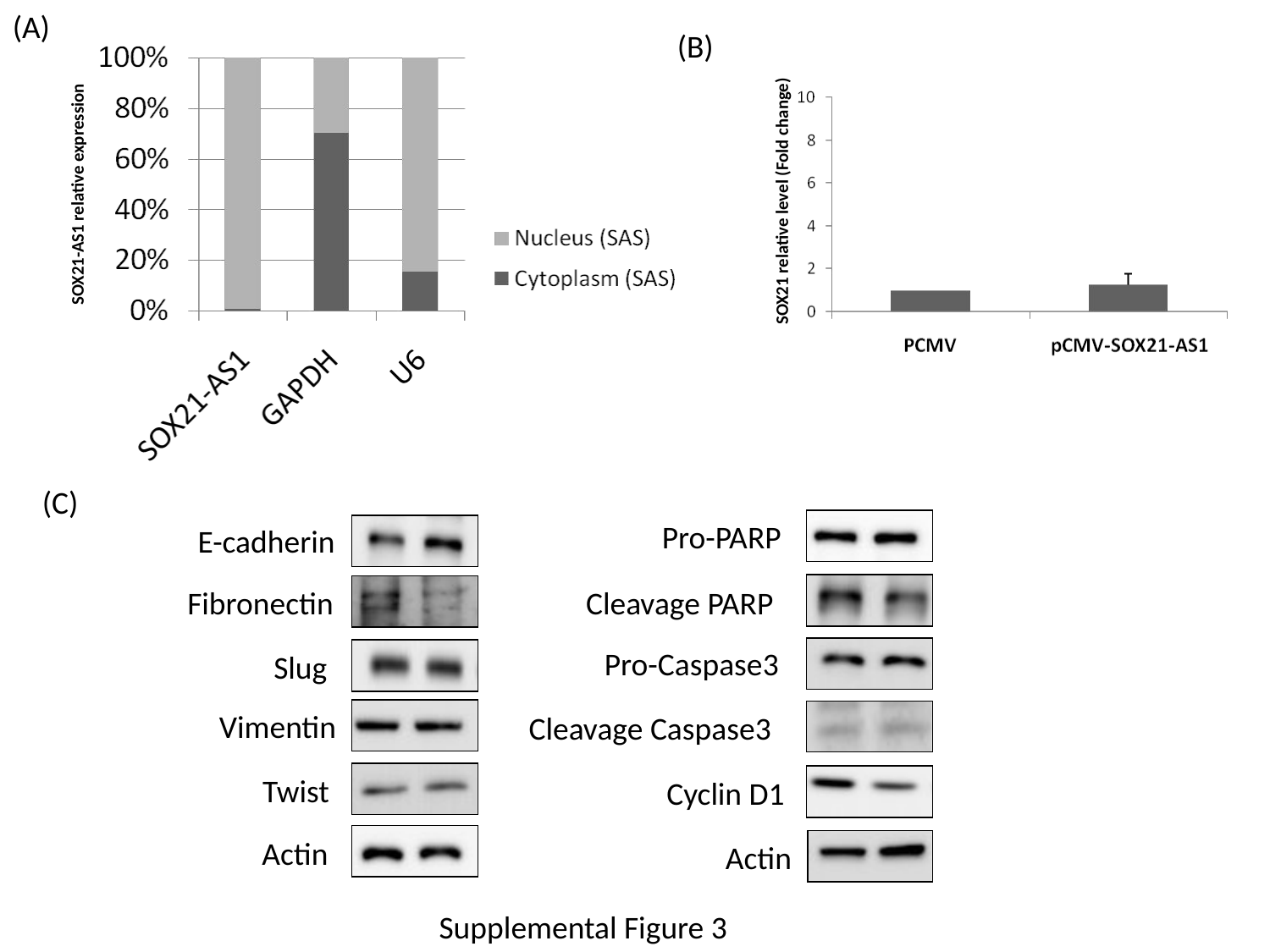

(A)
(B)
SOX21-AS1 relative expression
SOX21 relative level (Fold change)
(C)
Pro-PARP
E-cadherin
Fibronectin
Cleavage PARP
Pro-Caspase3
Slug
Vimentin
Cleavage Caspase3
Twist
Cyclin D1
Actin
Actin
Supplemental Figure 3
