# Supplementary material for: Aberrant DNA hypermethylation-silenced SOX21-AS1 gene expression and its clinical importance in oral cancer
Source: Clin Epigenetics. 2016 Nov 26;8:129. doi: 10.1186/s13148-016-0291-5 (PMC5124299; doi:10.1186/s13148-016-0291-5)
Supplement: Additional file 6: Table S2. — The detailed clinical characteristics of two OSCC patients. (DOC 29 kb) [file 13148_2016_291_MOESM6_ESM.doc]

| **Supplementary Table 2. The detailed clinical characteristics of two OSCC patients** | | | | | |
| --- | --- | --- | --- | --- | --- |
| Variables | Cell differentiation | T classification | N classification | M classification | AJCC pathological stage |
| Patient 1 | Moderate | T4 | N2 | M0 | IV |
| Patient 2 | Well | T1 | N0 | M0 | I |
|  | | | | | |
